# Anti-Inflammatory (M2) Response Is Induced by a sp^2^-Iminosugar Glycolipid Sulfoxide in Diabetic Retinopathy

**DOI:** 10.3389/fimmu.2021.632132

**Published:** 2021-03-18

**Authors:** Fátima Cano-Cano, Elena Alcalde-Estévez, Laura Gómez-Jaramillo, Marta Iturregui, Elena M. Sánchez-Fernández, José M. García Fernández, Carmen Ortiz Mellet, Antonio Campos-Caro, Cristina López-Tinoco, Manuel Aguilar-Diosdado, Ángela M. Valverde, Ana I. Arroba

**Affiliations:** ^1^ Research Unit, Instituto de Investigación e Innovación en Ciencias Biomédicas de la Provincia de Cádiz (INiBICA), Hospital Universitario Puerta del Mar, Cádiz, Spain; ^2^ Department of Endocrinology and Metabolism, University Hospital Puerta del Mar, Cádiz, Spain; ^3^ Department of Metabolism and Cell Signaling, Instituto de Investigaciones Biomédicas Alberto Sols (IIBm) (CSIC/UAM), Madrid, Spain; ^4^ Departamento de Química Orgánica, Facultad de Química, Universidad de Sevilla, Sevilla, Spain; ^5^ Instituto de Investigaciones Químicas (IIQ), CSIC - Universidad de Sevilla, Sevilla, Spain; ^6^ Área Genética, Dpto. Biomedicina Biotecnología y Salud Pública, Universidad de Cádiz, Cádiz, Spain; ^7^ Centro de Investigación Biomédica en Red de Diabetes y Enfermedades Metabólicas Asociadas (CIBERdem), ISCIII, Madrid, Spain

**Keywords:** sp^2^-iminosugar glycolipids, diabetic retinopathy, microglia, immunomodulation, M2 response

## Abstract

Diabetic retinopathy (DR) is one of the most common complications of Diabetes Mellitus (DM) and is directly associated with inflammatory processes. Currently, neuro-inflammation is considered an early event in DR and proceeds *via* microglia polarization. A hallmark of DR is the presence of retinal reactive gliosis. Here we report the beneficial effect of (*S*_S_,1*R*)-1-docecylsulfiny-5*N*,6*O*-oxomethylidenenojirimycin ((*Ss*)-DS-ONJ), a member of the sp^2^-iminosugar glycolipid (sp^2^-IGL) family, by decreasing iNOS and inflammasome activation in Bv.2 microglial cells exposed to pro-inflammatory stimuli. Moreover, pretreatment with (*Ss*)-DS-ONJ increased Heme-oxygenase (HO)-1 as well as interleukin 10 (IL10) expression in LPS-stimulated microglial cells, thereby promoting M2 (anti-inflammatory) response by the induction of Arginase-1. The results strongly suggest that this is the likely molecular mechanism involved in the anti-inflammatory effects of (*S*_S_)-DS-ONJ in microglia. (*S*_S_)-DS-ONJ further reduced gliosis in retinal explants from type 1 diabetic BB rats, which is consistent with the enhanced M2 response. In conclusion, targeting microglia polarization dynamics in M2 status by compounds with anti-inflammatory activities offers promising therapeutic interventions at early stages of DR.

## Introduction

Most patients with diabetes mellitus (DM) will develop some grade of diabetic retinopathy (DR) (1). The incidence of DR is growing and will increase several fold in the coming decades. It is the main cause of visual failure in the working-age population (2). DR is consider a common microvascular complication of DM, and it generally involves microvascular complications of capillary endothelium and pericytes leading to micro-aneurysm, blood retinal barrier (BRB) leakage and fragile new blood vessel formation (neovascularization) (3). Nevertheless, retinal neurodegeneration is present before to clinically-overt microvascular disturbances, and recent evidences suggest that neurodegeneration collaborates on the development of microvascular dysfunction and neovascularization (4). Several agents with neuroprotective actions are, therefore, being studied as potential therapeutics against DR (5).

**Figure 1:**
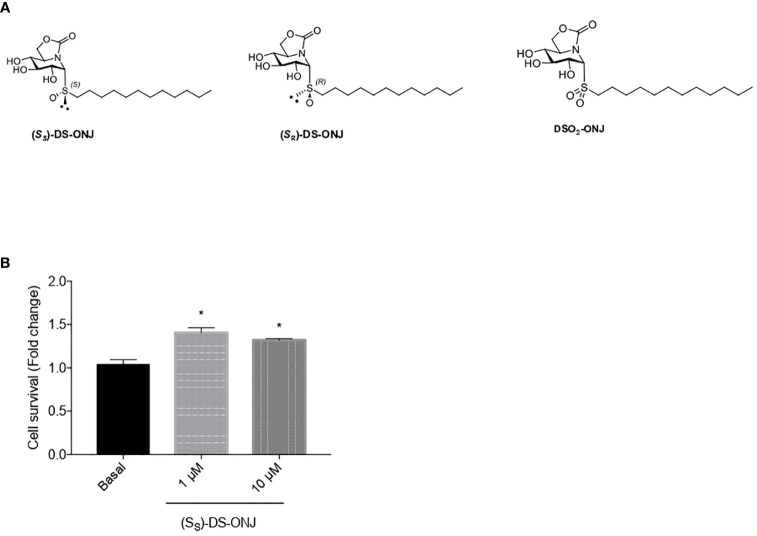
Effect of (*S*_S_)-DS-ONJ in the cellular viability in Bv.2 microglial cells. **(A)** Chemical structures of the sp^2^-IGLs (*S*_S_)-DS-ONJ, (*S*_R_)-DS-ONJ and DSO_2_-ONJ. **(B)** Bv.2 microglial cells were treated for 24 h with (*S*_S_)-DS-ONJ (1–10 μM) and viability was determined by crystal violet staining. Colorimetric quantification results presented as mean ± SEM (n = 5 independent experiments). The fold changes are presented relative to basal values. *p ≤ 0.05 *vs* the basal values (two-way ANOVA followed by Bonferroni t-test.)

Retinal inflammation is an early stage in DR, and plays a crucial role in its development (6). As occurs in other neurodegenerative diseases, DR displays an inflammatory component associated with alterations in the retina tissue. Inflammation released by increased glycolytic metabolites has been well characterized in patients with DR, and retinal inflammation contributes to vascular permeabilization and disruption of the BRB (7). Inflammation and apoptosis are important pathological processes of DR that often lead to necrosis of retinal cells (8). Consequently, compounds with inflammation and apoptosis regulatory capabilities have strong potential for the treatment of DR. In this context, we have previously observed that some sulfur-linked sp^2^-iminosugar glycolipids (sp^2^-IGLs), such as (1*R*)-1-dodecylsulfonyl-5*N*,6*O*-oxomethylidenenojirimycin (DSO_2_-ONJ) (**Figure 1**), efficiently promoted microglia polarization from the pro-inflammatory (M1) towards the anti-inflammatory (M2) state. Biochemical and computational data support a mechanism involving non-canonical auto-phosphorylation of p38α mitogen-activated protein kinase (MAPK), a key player in the inflammatory cascade, *via* binding of the sp^2^-IGL to the lipid binding site of the protein (6, 9). Contrary to other immuno-regulatory glycolipids, the sp^2^-IGLs are metabolically stable and can be prepared in the laboratory in pure anomeric form with total stereo-selectivity, thereby providing a suitable platform for glyco-drug design (10–16).

Available data on the biological activity of the sp^2^-IGLs reveal that the nature of the glycosidic group bridging the sugar-like glycone moiety (sp^2^-iminosugar) (17, 18) and the lipid aglycone plays a critical role in the anti-inflammatory potential, probably by favorably orienting the sp^2^-IGL molecule in the lipid binding pocket of p38α MAPK. Given the outstanding results obtained for DSO_2_-ONJ (9), replacing the sulfone tether by a closely-related quasi-isosteric functionality, e.g. a chiral sulfoxide group, seemed very promising in the search for new candidates for DR treatment. As a proof of concept, we have studied the effect of (*S*_S_,1*R*)-1-dodecylsulfinyl-5*N*,6*O*-oxomethylidenenojirimycin (referred to as (*S*_S_)-DS-ONJ, **Figure 1**) in Bv.2 microglial cells and retinal explants of type 1 diabetic BB rats within the environment of inflammation and immune modulation associated with DR. The data support the idea that (*S*_S_)-DS-ONJ induces of IL10 production and enhances of the action of arginase-1 in the M2 response, which is associated with inhibition of the inflammasome complex.

## Methods

### Reagents

Fetal bovine serum (FBS) and culture media were obtained from Invitrogen (Grand Island, NY, USA). Bovine serum albumin (BSA), MTT, crystal violet, glutaraldehyde, N-(1-naphthyl) ethylenediamine (NEDA), sulfanilamide and bacterial lipopolysaccharide (LPS) were purchased from Sigma-Aldrich (St Louis, MO, USA). IL4 and IL13 were purchased from Preprotech (London, UK). Acrylamide, immunoblot PVDF membranes were purchased from Bio-Rad (Madrid, Spain). BCA reagent and chemiluminescent HRP substrate were purchased from Pierce (Rockford, IL, USA).

### Antibodies

Antibodies against IκBα (sc-371), JNK (sc-571), p65-NFκB (p65) (sc-732) and phospho-p38α MAPK (Thr 180/Tyr182) (sc-17852-R) were purchased from Santa Cruz Biotechnology (Palo Alto, CA, USA). Anti-phospho JNK (Thr183/Tyr185) (#4668) and anti-p38α MAPK (#9212) antibodies were purchased from Cell Signaling Technology (Danvers, MA, USA). Anti-caspase-1 (p10) (ab 179515), iNOS (ab 15323), HO-1 (ab 137749), antibodies were purchased from Abcam (Cambridge, UK). Anti-Arginase-1 (BD610708) antibody was purchased from BD Bioscience (Madrid, Spain). Anti-Glial Fibrillary Acidic Protein (GFAP) antibody (Z0334) was obtained from DAKO (Glostrup, Denmark). Anti-NLRP3 (AG-20B-0006) was purchased from AdipoGen Life Science (Liestal, Switzerland) and anti-α-tubulin (T-5168) antibody was from Sigma-Aldrich (St Louis, MO, USA).

### Synthesis of the sp^2^-Iminosugar Glycolipid (*S*_S_)-DS-ONJ

(*S*_S_1*R*)-1-Dodecylsulfinyl-5*N*,6*O*-oxomethylidenenojirimycin (referred to as (*S*_S_)-DS-ONJ) was synthesized by controlled oxidation of (1*R*)-1-dodecylthio-5*N*,6*O*-oxomethylidenenojirimycin with *m*-chloroperbenzoic acid and subsequent column chromatography separation from the corresponding (*S*_R_)-diastereomer (*S*_R_)-DS-ONJ (**Figure 1A**), following the procedure previously reported (16).

### Cell Culture

Murine microglia Bv.2 cell line was generously provided by Dr. M. L. Nieto (IBGM, Spain) and previously has been published [6,9,14]. Bv.2 cells were cultured at 37°C in a humidified atmosphere with 5% CO_2_ in RPMI supplemented with 10% (v/v) heat inactivated FBS, 1% (v/v) penicillin/streptomycin (Sigma) and 2 mM L-glutamine (Gibco, Carlsbad, California, USA). Bv.2 cells were grown to 70% confluence and then washed twice with PBS. The cells were further cultured in serum-free medium and subsequently stimulated with LPS (200 ng/mL) in the presence or absence of (*S*_S_)-DS-ONJ (1, 10, 50 μM) for 24h in cell survival and Griess test approaches. In further experiments, Bv.2 were pre-incubated with (*S*_S_)-DS-ONJ at 10 μM or a mixture of IL4/IL13 (10ng/mL each; a M2 response inductors) for 4 h before the addition of LPS (200 ng/mL) for a further 24 h.

### Retinal Explants

All animal procedures were performed with the approval of the Cádiz University School of Medicine (Cádiz, Spain) Committee for the Ethical Use and Care of Experimental Animals. Animal experimentation conducted in this study followed the recommendations of the Federation of European Laboratory Animal Science Associations (FELASA) on health monitoring in accordance with the regulations of the Association for Research in Vision and Ophthalmology (ARVO).

Bio-Breeding (BB) and Wistar rats were maintained under conventional conditions in an environment-controlled room (20-21°C, 12 h light-dark cycle) with water and standard laboratory rat chow available *ad libitum*. Blood samples from the tail vein was used in BB rats for weekly random glucose measurements using an automatic glucose monitor (Freestyle Optium Neo, Abbott, Madrid, Spain). Diabetes onset was defined by glucose levels above 270 mg/dL (14.98 mmol/L). *In vivo* assays were performed with retinas from 6-week-old male or female Wistar and BB rats. The rats were euthanized by an overdose of anesthesia, and the eyes were enucleated. The lens, anterior segment, vitreous body, retinal pigment epithelium and sclera were removed. The retinas were immediately frozen for protein extraction. The animals were sacrificed at 4, 5 or 6 weeks of age according to the experimental approach.

*Ex vivo* assays were performed with retinas from 6-week-old male or female BB rats. The retinas from BB rats were cultured in R16 medium (provided by Dr. P.A. Ekstrom, Lund University, Sweden) with no additional serum. Retinas were cultured with (*S*_S_)-DS-ONJ at 20 μM as indicated in the figure legends.

### Analysis of the Cellular Viability by Crystal Violet Staining and MTT Assay

After cell treatments for 24 h, the medium was discarded and the remaining viable adherent cells were fixed with 10% glutaraldehyde and stained with crystal violet (0.1% w/v in water) for 20 min. The plates were then rinsed with tap water and allowed to dry. Acetic acid (10%) was added to solubilize the crystal violet. The absorbance of each plate was read spectrophotometrically at 590 nm. In order to corroborate the lack of cytotoxic effects on Bv.2 cells, a MTT assay (Merck KGaA, Darmstadt, Germany) was performed. Briefly, MTT (5 mg/mL) was dissolved in PBS and filter sterilized. Then, 100 μL of the prepared solution was added to each well. This was incubated until purple precipitate was visible. Subsequently, 100 µL of DMSO was added to each well and incubated in darkness for 2 h at room temperature. The absorbance was measured at a wavelength of 595 nm.

### Analysis of Nitrites (NO_2_^−^)

Concentrations of NO_2_^−^ were measured using the Griess method (19) at the time defined in cell culture section. Briefly, nitrites turn into pink color in contact with an acid solution containing 1% sulfanilamide and 0.1% N-(1-naphthyl) ethylenediamine (NEDA). The resulting pink colour is quantified by colorimetry at 540 nm in a microplate reader (PowerWave, Bioteck, Torino, Italy).

### Immunofluorescence

Bv.2 microglial cells were seeded on coverslips 24 h before LPS stimulation and/or (*S*_S_)-DS-ONJ in serum-free medium. Then, cells were washed in PBS, fixed with 4% (w/v) paraformaldehyde in PBS for 10 min at room temperature, washed in PBS and permeated with 0.4% (v/v) Triton X-100 in PBS for 20 min. Blocking in PBS containing 3% (w/v) BSA and 0.1% (v/v) Triton X-100 for 2 h and the cells were then left overnight in a humid chamber at 4°C with rabbit anti-p65 NFκB antibody (1:1000) in blocking buffer (TBS containing 3% (w/v) BSA and 1% (v/v) Triton X-100). After that, the cells were incubated in the dark for 2 h with anti-rabbit conjugated Alexa 488 antibody (Molecular Probes, ThermoFisher Scientific, Waltham, MA, USA). The nucleus were stained with 4,6-diamidino-2-phenylindole (DAPI) and mounted with Fluoromount G medium.

For immunofluorescence analysis, the retinal explants were fixed in 4% (w/v) paraformaldehyde for 24 h at 4°C. Then, the whole retina was washed in TBS containing 0.1% (w/v) BSA and 0.1% (v/v) Triton X-100 (this buffer was used for all subsequent washes), and blocked and permeated for 2 h in TBS containing 3% (w/v) BSA and 1% (v/v) Triton X-100. Subsequently, the retinal explants were then incubated overnight in a humid chamber at 4°C with rabbit anti-GFAP antibody (1:1000) in the blocking solution. Retinal explants were washed and incubated for 90 min with anti-rabbit immunoglobulin antibody conjugated to Alexa 488 (1:2000; Molecular Probes, Eugene, OR, USA). After washing, retinal explants were mounted with medium (Fluoromount G) containing DAPI. Staining was observed and recorded with an inverted laser confocal microscope Fluoview MPE/FV 1000, Olympus, MA, USA).

### Quantitative Real-Time Polymerase Chain Reaction (qPCR) Analysis

Total RNA was extracted with Trizol^®^ reagent (Invitrogen, Madrid, Spain) and reverse transcribed using a SuperScript™ III First-Strand Synthesis System for qPCR following the manufacturer’s recommendations (Invitrogen). qPCR was performed with a Corbett Rotor-Gene 6000, Qiagen sequence detector. Primer-probe sets for mouse *Tnfa, Il6, Il1b, Il10, Nlrp3, Nos2, Arg1* and *18s* were purchased from Applied Biosystems.

### Western Blot

Proteins were resolved using denaturing SDS-PAGE, and transferred to PVDF membranes (Bio-Rad). Membranes were blocked using 5% nonfat dried milk or 3% BSA in 10 mM Tris-HCl, 150 mM NaCl, pH 7.5 (TBS), and incubated overnight with several antibodies (1:1000 unless otherwise stated) in 0.05% Tween-20-TBS. Immunoreactive bands were visualized using the enhanced chemiluminescence reagent (Bio-Rad).

### Statistical Analysis

Western blot quantification was performed using the ImageJ program. Values in all graphs are presented as means ± SEM. Statistical tests were performed using the SPSS 21.0 package for Windows (SPSS Inc. IBM, Armonk, NY, USA). Data were analyzed using one-way ANOVA followed by Bonferroni test or Student paired *t*-test when comparisons were between any two groups. Differences were considered significant at p ≤ 0.05.

## Results

### Anti-Inflammatory Effects of (*S*_S_)-DS-ONJ in LPS-Stimulated Bv.2 Microglial Cells

Chemical structures of sp^2^-IGLs (*S*_S_)-DS-ONJ, (*S*_R_)-DS-ONJ and DSO_2_-ONJ are presented in **Figure 1A**. First, we tested the cytotoxicity of (*S*_S_)-DS-ONJ (**Figure 1B**) on microglia Bv.2 cells at various concentrations (**Figure 1B**; **S1A, B**). Based on these results we used 1 and 10 μM concentrations for subsequent experiments.

**Figure 2:**
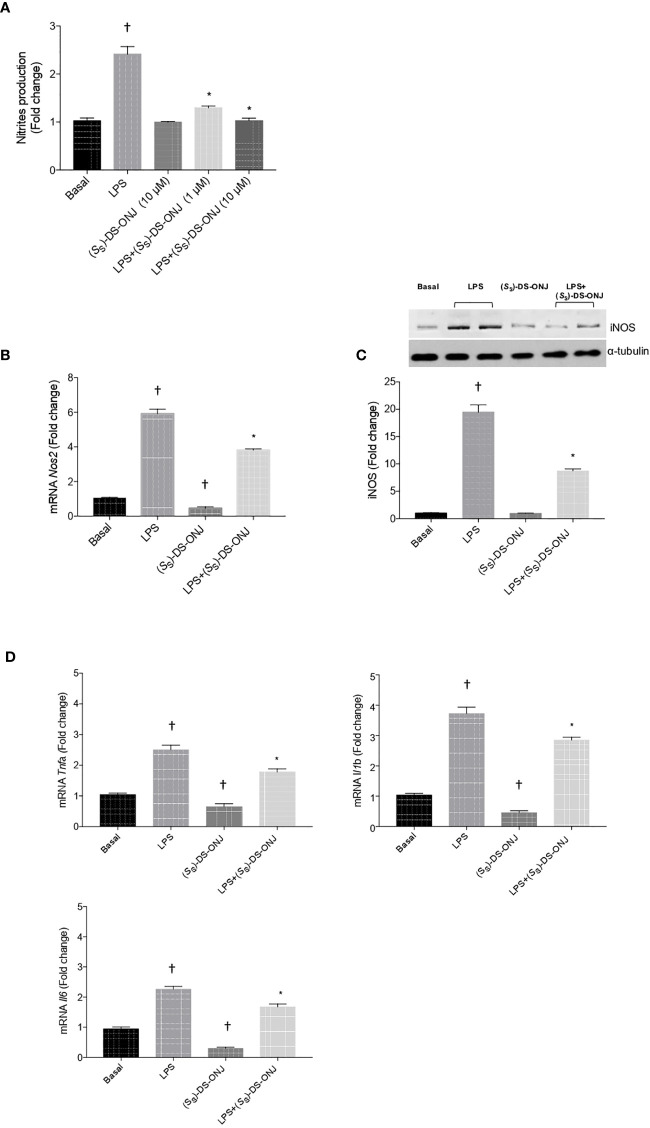
Protective effects of (*S*_S_)-DS-ONJ against LPS-mediated iNOS activation and elevation of mRNA levels of pro-inflammatory cytokines in Bv.2 microglial cells. Bv.2 microglial cells were treated for 24 h with LPS (200 ng/mL) or LPS plus (*S*_S_)-DS-ONJ (1–10 μM). **(A)** Colorimetric quantification of nitrites was performed. **(B)** mRNA of *Nos2* was determined by qRT-PCR. **(C)** Protein extracts were analyzed by Western blot with the corresponding antibodies against iNOS and α-tubulin as loading control. Representative autoradiograms are shown. Blots were quantified by scanning densitometry and the results are presented as mean ± SEM. **(D)** *Tnfa, Il1b*, and *Il6* mRNA values were determined by qRT-PCR. The results are presented as means ± SEM (n = 6 independent experiments). Fold changes are calculated relative to the basal value. *p ≤ 0.05 *vs* LPS treatment, ^†^p ≤ 0.05 *vs* basal value (two-way ANOVA followed by Bonferroni t-test.).

For more insight on the effects of (*S*_S_)-DS-ONJ in microglia, Bv.2 cells were stimulated with LPS, a classical M1 stimulus, after pre-treatment with (*S*_S_)-DS-ONJ for 4 h. The LPS stimulation induced a pro-inflammatory environment similar to that found in the diabetic environment (6, 9, 20). Classically activated or M1 immune cells (macrophages and microglial cells), which are pro-inflammatory and polarized by lipopolysaccharide (LPS), produce pro-inflammatory cytokines such as interleukin1β, IL6, and TNFα (21). Bv.2 microglial cells were cultured for 24 h in the presence or absence of LPS (200 ng/mL) plus (*S*_S_)-DS-ONJ (1 or 10 μM) or M2 cytokines (IL4/IL13) in the pre-treatment regimen. As shown in **Figure 2A** and **S1C**, the elevation of nitrites in the culture medium induced by LPS was significantly reduced by the pre-treatment with (*S*_S_)-DS-ONJ in a dose-dependent manner. The 10 μM concentration of (*S*_S_)-DS-ONJ showed the highest anti-inflammatory effect, counteracting LPS-stimulation. Similarly, *Nos2* mRNA and iNOS protein concentrations, which were elevated in LPS-treated Bv.2 microglial cells, significantly decreased in the presence of (*S*_S_)-DS-ONJ (**Figures 2B, C**). The effects of (*S*_S_)-DS-ONJ on the mRNA levels of pro-inflammatory cytokines in Bv.2 microglial cells were studied. As depicted in **Figure 2D**, the significant increases in *Tnfa, Il1b* and *Il6* mRNAs induced by LPS stimulation were ameliorated by the pre-treatment with (*S*_S_)-DS-ONJ. **Supplementary Figure 2** shows that the effect of IL4+IL13 cytokines evidenced by the decrease of LPS-induced mRNA levels of TNFα, IL1β, IL6 and *Nos2* was also evident.

### The Classical Pro-Inflammatory Kinases Stress-Activated Pathways Are Modulated by (*S*_S_)-DS-ONJ

**Figure 3:**
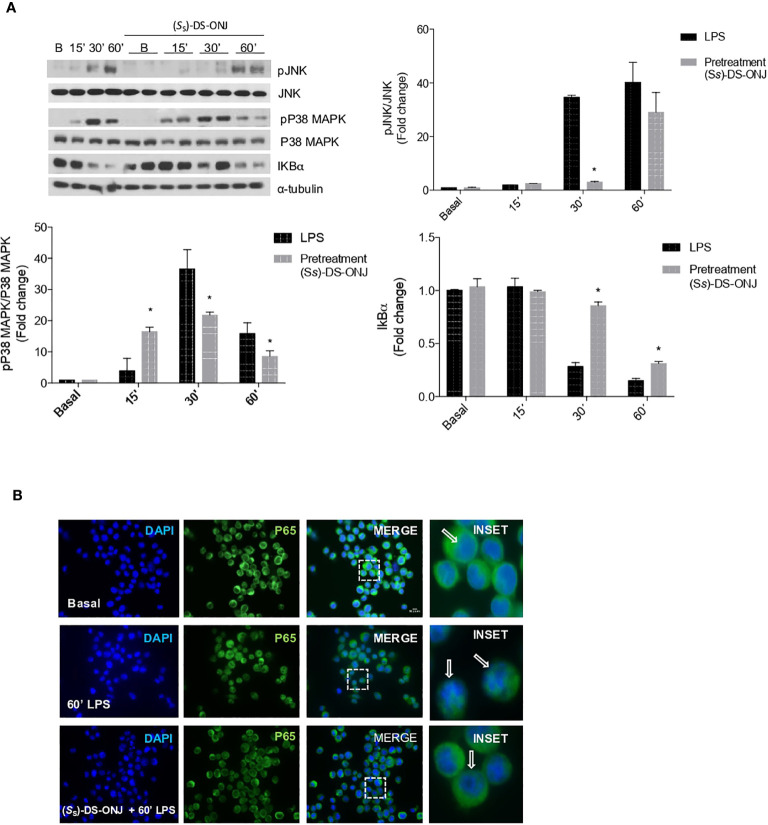
Effects of (*S*_S_)-DS-ONJ in the activation of MAPKs and NFκB-mediated signaling in LPS-stimulated Bv.2 microglial cells. Bv.2 microglial cells were stimulated with 200 ng/mL LPS in the absence, or presence, of 10 μM (*S*_S_)-DS-ONJ for the indicated time periods. **(A)** Protein extracts were separated by SDS-PAGE, and analyzed by Western blot with antibodies against phosphorylated (p)-JNK, total JNK, phosphorylated (p)-p38α MAPK, total p38α MAPK, IκBα and α-tubulin. Representative autoradiograms are shown (n = 7 independent experiments). Blots were quantified with scanning densitometry, and the results are presented as mean ± SEM. The ratios between the indicated proteins and the fold changes relative to the basal values are shown. *p ≤0.05 *vs* LPS treatment (two-way ANOVA followed by Bonferroni t-test). **(B)** Confocal immunofluorescence assessment of the nuclear translocation of p65-NFκB in Bv.2 microglial cells following stimulation with LPS in the presence or absence of (*S*_S_)-DS-ONJ. Activation of p65-NFκB nuclear translocation was defined by an increase in immunofluorescence of p65-NFκB (green channel) in the nuclear regions. Nuclear regions of Bv.2 microglial cells were determined by counterstaining nuclear DNA with DAPI (blue channel). White arrows indicate the p65-NFκB nuclear o cytoplasmic localization.

The classical MAPKs and NFκB-mediated signaling pathways involved in the inflammatory processes were examined. LPS-stimulation induced a rapid, and maximal, effect at 30 min in the phosphorylation of JNK and p38α MAPK, in parallel with IkB degradation (**Figure 3A**). Moreover, treatment with (*S*_S_)-DS-ONJ exerted an anti-inflammatory effect by inducing a decrease in the phosphorylation of JNK, and prevented LPS-mediated degradation of IkB. In addition, the pre-treatment with (*S*_S_)-DS-ONJ promoted significantly higher levels of p38α MAPK phosphorylation, as compared to LPS stimulus alone (**Figure 3A**).

Previous results obtained with the sulfone analogue DSO_2_-ONJ indicated that sp^2^-IGLs induced local conformational changes in p38α MAPK, leading to its auto-phosphorylation and activation (9, 14). Pre-treatment with (*S*_S_)-DS-ONJ prevented LPS-mediated nuclear translocation of p65-NF-κB (**Figure 3B**).

### (*S*_S_)-DS-ONJ Reduces the LPS-Induced Inflammation by Inflammasome Inhibition in Bv.2 Microglial Cells

Since in LPS-stimulated microglia IL1β is processed *via* caspase-1 through the NACHT, LRR and PYD domains-containing protein 3 (NLRP3) inflammasome complex (22), we analyzed whether the anti-inflammatory effect of (*S*_S_)-DS-ONJ was dependent on the inflammasome activation.

**Figure 4:**
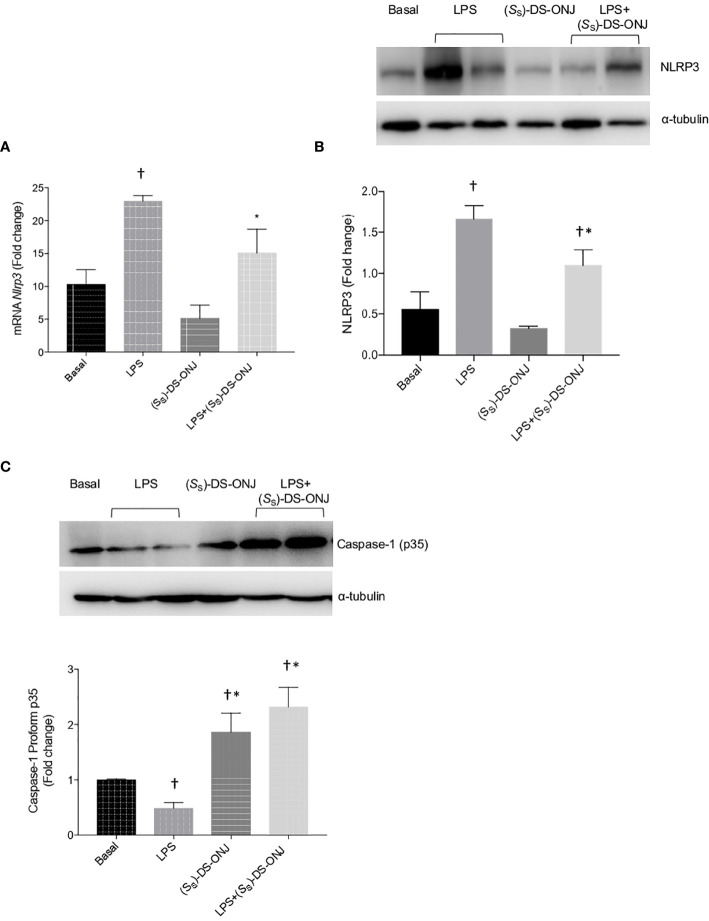
Protective effects of (*S*_S_)-DS-ONJ against LPS-mediated inflammasome activation in Bv.2 microglial cells. **(A)** *Nlrp3* mRNA was measured by qRT-PCR. **(B)** Protein extracts were analyzed by Western blot, with antibodies against NLRP3; α-tubulin was used as loading control. **(C)** Protein extracts were analyzed by Western blot, with antibodies against caspase-1; α-tubulin was used as loading control. Representative autoradiograms are shown (n = 6 independent experiments). Blots were quantified using scanning densitometry, and the results are presented as means ± SEM. The results are presented as means ± SEM (n = 6 independent experiments). Fold changes are calculated relative to the basal values. *p ≤ 0.05 *vs* LPS treatment, ^†^p ≤ 0.05 *vs* basal values (two-way ANOVA followed by Bonferroni t-test).

The mRNA and protein levels of Nlrp3 were markedly increased after LPS stimulation. Such levels were significantly reduced upon treatment with (*S*_S_)-DS-ONJ (**Figures 4A, B**). **Figure 4C** shows the accumulation of pro-caspase-1 pro-form (unprocessed form) in response to (*S*_S_)-DS-ONJ in Bv.2 microglial cells.

### M2 Anti-Inflammatory Response Is Promoted by (*S*_S_)-DS-ONJ in Bv.2 Microglial Cells

**Figure 5:**
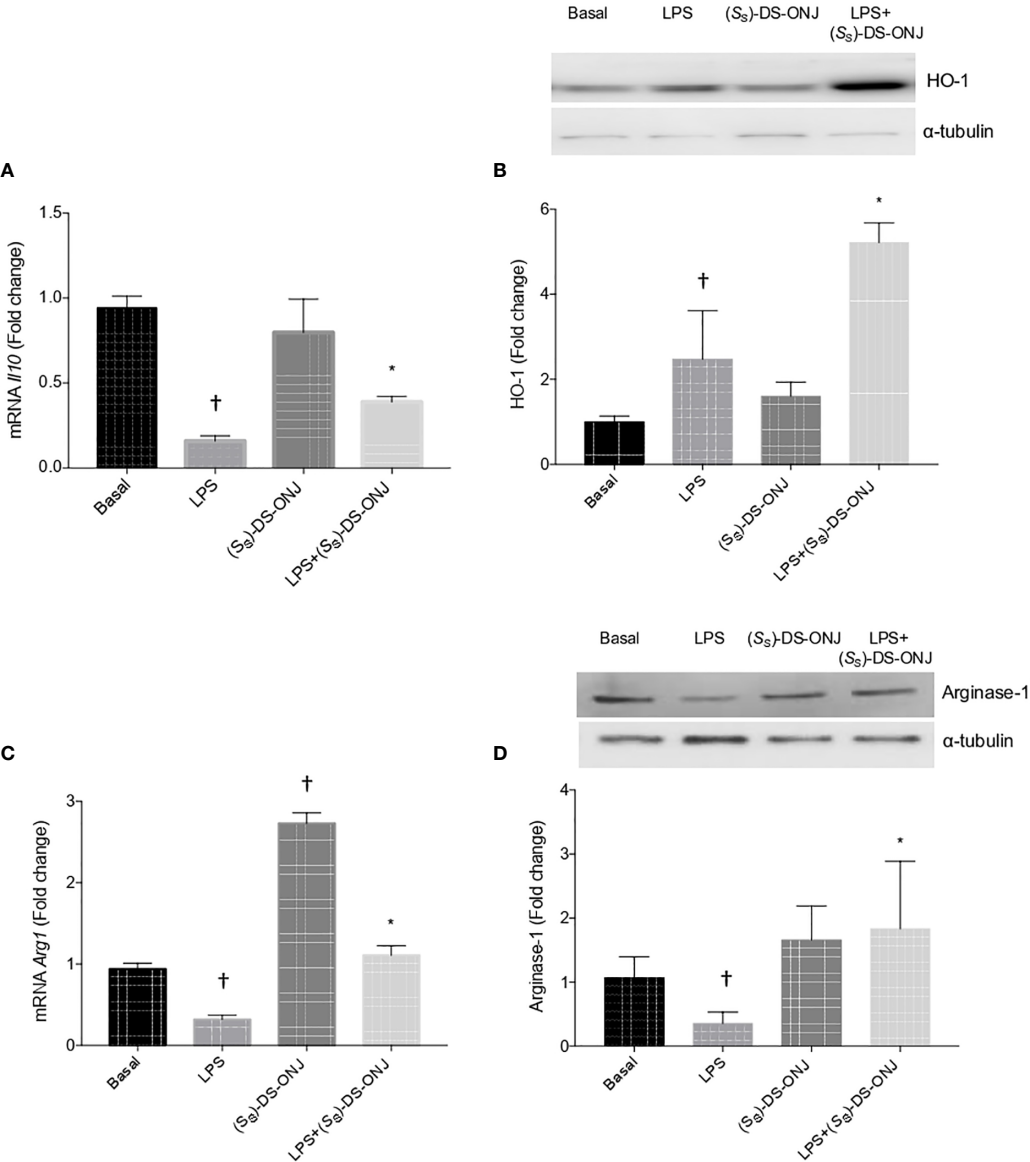
The anti-inflammatory response mediated by HO-1 and IL-10. Bv.2 microglial cells were stimulated with 200 ng/mL LPS in the presence, or absence, of 10 μM (*S*_S_)-DS-ONJ for 24 h. **(A)** *Il10* mRNA determined by qRT-PCR. **(B)** Protein extracts were analyzed by Western blot with antibodies against HO-1 and α-tubulin (loading control). Representative autoradiograms are shown. Blots were quantified using scanning densitometry. **(C)** *Arg1* mRNA determined by qRT-PCR. The results are presented as means ± SEM. **(D)** Protein extracts were analyzed using Western blot, with antibodies against Arginase-1; α-tubulin was a loading control. Representative autoradiograms are presented. Blots were quantified using scanning densitometry. The results are presented as means ± SEM. Fold changes are relative to the basal values. *p ≤ 0.05 *vs* LPS treatment, ^†^p ≤ 0.05 *vs* the basal values (two-way ANOVA followed by Bonferroni t-test).

To evaluate this aspect further, we determined mRNA *Il10*, an anti-inflammatory cytokine. As shown in **Figure 5A**, LPS-stimulation induced a significantly decrease in *Il10* mRNA, and this effect was abolished with (*S*_S_)-DS-ONJ treatment. Moreover, (*S*_S_)-DS-ONJ induced an increase in HO-1 protein concentrations, an anti-oxidant molecule also with anti-inflammatory effects (**Figure 5B**).

We evaluated Arginase-1 expression as a marker of M2 phenotype of microglia in Bv.2 microglial cells stimulated with LPS with or without (*S*_S_)-DS-ONJ pretreatment. **Figures 5C, D** summarizes the results showing that (*S*_S_)-DS-ONJ induced an increase in mRNA, and protein expression, of Arginase-1 either on its own, or in the presence of LPS.

### Retinal Gliosis Is Reduced in Retinal Explants From BB Rats Treated With (*S*_S_)-DS-ONJ

**Figure 6:**
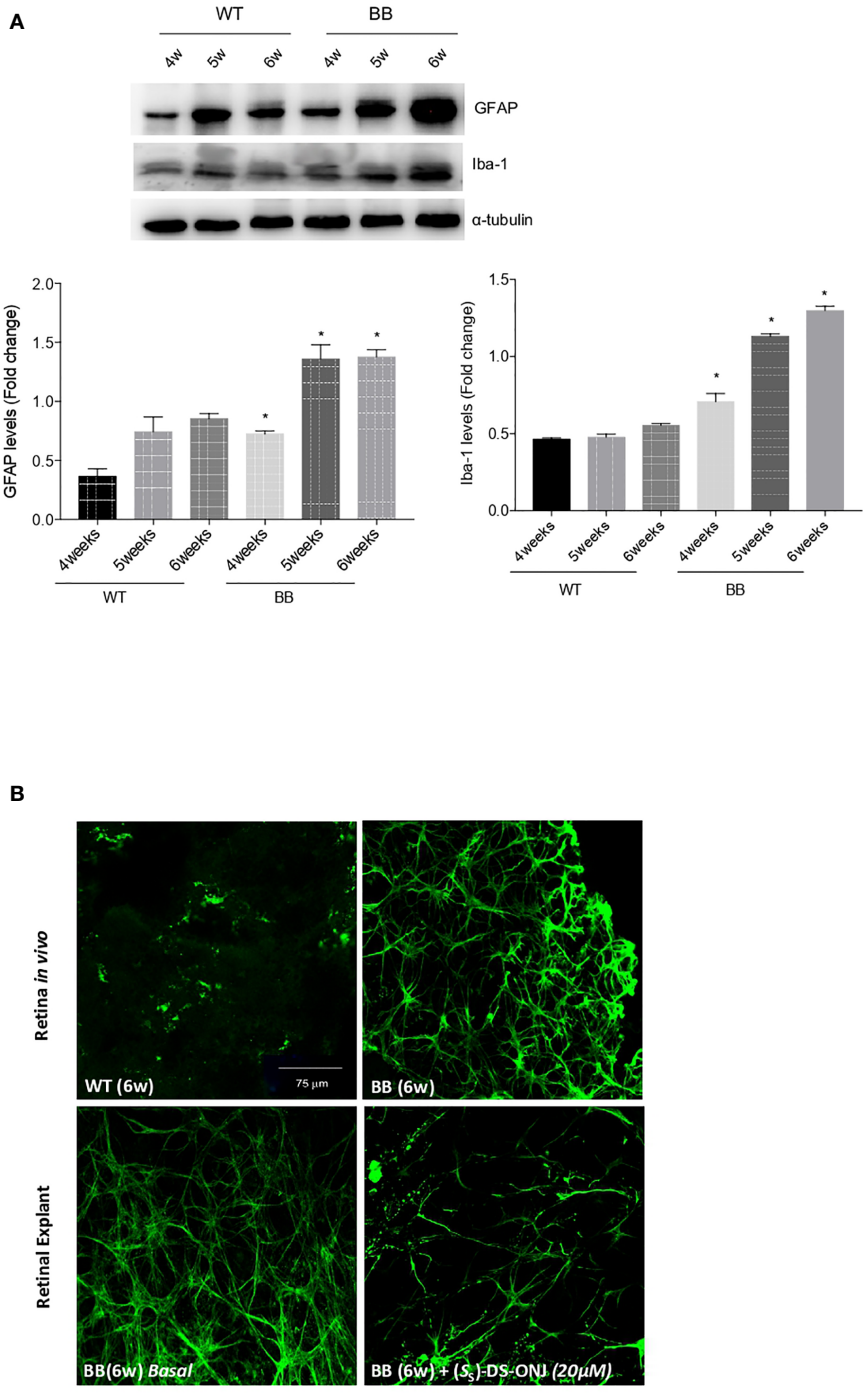
Reactive gliosis is present in BB rats from 6 weeks of age, and treatment with (*S*_S_)-DS-ONJ reduced reactive gliosis. **(A)** Analysis of GFAP and Iba-1 in retinas from BB rats during DR progression. Protein concentrations in retinal extracts were analyzed by Western blot, with α-tubulin used as loading control. Representative autoradiograms are shown. Results are presented as means ± SEM (n = 5 retinas per condition). *p ≤ 0.05 BB *vs* WT matched at each age. **(B)** (upper panels) Retinas from 6 weeks old BB and WT rats. (lower panels) Retinal explants from 6 weeks old BB rats were treated for 24 h with (*S*_S_)-DS-ONJ (20 μM) or treatment vehicle alone. Immunostaining for GFAP (green) was performed in whole retinas. Representative images are shown (n = 5 retinas per condition).

To establish the time-frame in which BB rats exhibit clear pro-inflammatory parameters associated with DR, we analyzed the changes in the concentrations of GFAP in a time-course experiment. GFAP is a marker of gliosis, which is a hallmark of neuro-degenerative retinal diseases, including DR (23, 24). Reactive GFAP is present in the retinas of BB rats at 4 weeks of age, and continue to increase in the following weeks (**Figure 6A**). To quantify the inflammatory environment present during the early stages of DR, we determined the specific reactive microglia marker Iba-1. We observed that values were elevated in the retinas from BB rats at 4 to 6 weeks of age and increased in a time-dependent manner (**Figure 6A**).

We used BB rats as an animal model of autoimmune T1DM, and observed that the expression of reactive gliosis was detectable in the retinas of rats at 6 weeks of age (**Figure 6B**). Our previous results in *db/db* mice (6, 9) had shown that DR features were maintained in retinal explants. Hence, we prepared retinal explants from 6 weeks-old rats that were treated with (*S*_S_)-DS-ONJ at 20 μM for 24 h; this dose was judged optimal after previous dose-response study (data not shown). The analysis of GFAP expression by immunofluorescence revealed a dramatic blunting of reactive gliosis in retinal explants treated with (*S*_S_)-DS-ONJ (**Figure 6B**).

## Discussion

Most of the new therapeutic approaches to combat DR are directed towards the immune system. Indeed, the pro-inflammatory environment in the course of DR is associated with altered immune responses (6) in the retina and, therefore, their modulation offers appealing opportunities to slow DR progression, and prevent the loss of visual function.

In this study, we provide new mechanistic evidence on the anti-inflammatory activity of the (*S*_S_)-DS-ONJ member of sp^2^-IGLs, using an *in vitro* cellular bioassay that reproduces the pro-inflammatory environment of DR. In previous studies, we demonstrated that different sp^2^-iminosugar glycolipids (25) elicit beneficial effects due to their anti-inflammatory potential. The results had revealed that the nature of the glycosidic group bridging the sugar-like glycone moiety and the lipid aglycone confers the ability to exert an anti-inflammatory effect by triggering different signaling pathways in microglia. Of relevance is that the sulfone DSO_2_-ONJ elicits anti-inflammatory effects, probably by favorably orienting the sp^2^-IGL molecule in the lipid binding pocket of p38α MAPK (9). In this regard, we recently demonstrated (9) that the (*S*_R_)-configured sulfoxide (*S*_R_)-DS-ONJ attenuates JNK and NFκB signaling pathways. In this study, we extended the research into the effects of the related sulfoxide (*S*_S_)-DS-ONJ. Here as well, we found potent anti-inflammatory effects preventing LPS-induced stress in Bv.2 microglial cells as well as retinal explants from type 1 diabetic BB rats. A further step investigated the molecular mechanisms involved.

Treatment of Bv.2 microglial cells with a non-toxic concentration (10 µM) of (*S*_S_)-DS-ONJ or IL4/IL13 mixture reduced NO production and inhibited LPS-induced *Nos2* and pro-inflammatory cytokines mRNA expression. This result corroborates that (*S*_S_)-DS-ONJ presents anti-inflammatory effects and induces an M2-response similar to those observed for the classical M2-mediated cytokines stimulus. Importantly, (*S*_S_)-DS-ONJ increased the *Il10* mRNA, an anti-inflammatory cytokine. The detailed study of the pro-inflammatory signaling pathways involved in these effects showed that (*S*_S_)-DS-ONJ successfully inhibited LPS-mediated degradation of IκB, and the translocation of p65 NFκB to the nucleus, as the analogues sp^2^-IGL molecules (DSO_2_-ONJ and (*S*_R_)-DS-ONJ) did. Moreover, (*S*_S_)-DS-ONJ targeted the inflammasome complex by blocking *Nlrp3* mRNA expression. Of note, the accumulation of pro-form caspase-1 by (*S*_S_)-DS-ONJ and the decrease of *Nlrp3* mRNA expression and protein levels could explain the observed inflammasome complex inhibition. In this regard, the (*S*_S_)-DS-ONJ treatment is able to modulate several key targets in the inflammatory processes, while driving towards a delayed pro-inflammatory response and promoting the anti-inflammatory response.

The mechanisms by which immuno-regulatory sp^2^-IGLs increase Arginase-1 and IL10 in microglia are, still, only partially understood. Microglia cells show specific phenotypes and are modified depending on the activation signals and the cytokine environment to which they are exposed. The M2 microglial cells phenotype arises in the presence of the anti-inflammatory cytokine IL10 (26) which could increase Arginase-1 expression, as detected in our study. By contrast, no changes in the levels of other anti-inflammatory cytokines expression, such as IL4 or IL13, were detected upon (*S*_S_)-DS-ONJ treatment (data not shown). The anti-inflammatory cocktail of both IL4 and IL13 cytokines produces a strong protective response against the effect of LPS; however, our results show that (*S*_S_)-DS-ONJ treatment does not modulate IL4 and/or IL13 expression.

Up-regulation of HO-1, an anti-oxidant molecule, is an essential cyto-protective mechanism activated in response to cellular insult. Moreover, HO-1 is able to mediate a robust anti-inflammatory effect through of inhibition of pro-inflammatory cytokines production by activated macrophages (27). Conversely, blocking HO-1 expression reduces the M2 markers IL10 and Arginase-1 (28). In this study, we demonstrated a direct link between the induction of HO-1 protein expression and the increase in *Il10* and *Arg1* mRNA in Bv.2 microglial cells treated with (*S*_S_)-DS-ONJ. The anti-inflammatory action of (*S*s)-DS-ONJ has been connected to critical nodes of different signaling pathways involved. We hypothesized that these effects could be synergistic, concertedly generating a strong anti-inflammatory response in microglial cells.

Our results are in agreement with the classical cellular response to an acute insult in which cellular defenses are activated only in response to a deleterious environment. As demonstrated in recent studies, HO-1 and IL10 are able to induce their anti-inflammatory effects in a reciprocal manner (29). Also, it is important to note that this reciprocal induction is dependent on p38α MAPK activation (30, 31). We previously demonstrated that DSO_2_-ONJ induced p38α MAPK auto-phosphorylation upon binding to the allosteric lipid binding site, a conformational structural change. Also, we observed a similar effect in the presence of (*S*_S_)-DS-ONJ. Of note is that both compounds share the same lipid chain, and differ only in the oxidation state of the sulfur atom (sulfone and sulfoxide, respectively; **Figure 1A**).

As with most diabetic complications, DR is a multifactorial disease that is linked to inflammation, hyperglycemia and neuronal dysfunction, as main features in its pathogenesis (32). T1DM is defined as a chronic, low-grade, systemic, inflammatory disease characterized by changes in the secretion of cytokines as well as the polarization of tissue-resident immune cells. Innate immune microglial-cells in the central nervous system tend towards the M1 state (33), as demonstrated by the increased plasma concentrations of pro-inflammatory cytokines (34). The BB rat is considered a good animal model for DR study because it mimics human DR progression and, hence, this animal model has been used to analyze processes such as retinal neurodegeneration and retinal vascular leakage, as well as to study potential pharmacological treatment options in preclinical studies (35, 36). With respect to diabetes-associated retinal inflammation, a previous study in different animal models described an increase in inflammatory markers in the retinal tissue (37, 38). In the present study, we used BB rats to explore the switching from M1 towards M2 stage during DR in a systemic pro-inflammatory environment. Our data showed that GFAP and Iba-1, classic markers of activated microglia cells, increased in an age-dependent manner in the retina of diabetic BB rats. Of relevance is that reactive gliosis was evidenced by increased GFAP expression in BB rats at 6 weeks of age, with patterns of Mueller cells foot swelling processes (39) associated with cell death, or apoptosis (40).

Our previous data in retinal explants from *db/db* mice demonstrated that treatment with the sulfone sp^2^-IGL DSO_2_-ONJ ameliorated reactive gliosis (6, 9). Interestingly, reactive gliosis in retinal explants from BB rats, a T1DM autoimmune animal model, was also reduced by the treatment with the sulfoxide analogue (*S*_S_)-DS-ONJ, showing a regression towards an early stage of DR. As reactive gliosis is present in DR (40–42), these results might have new therapeutic implications such as novel strategies for targeting DR associated with T1DM or T2DM based on the specific induction of the M2 response in the retina through sp^2^-IGL-mediated increase in Arginase-1. The analogues of sp^2^-IGLs, with specific structural modifications, share a common anti-inflammatory response mediated by different signaling cascades. As such, a combination of these analogues could show synergistic effects in the neuro-inflammatory context. Similarly, the strong M2 response induced in diabetic retinal inflammation models offers the possibility of extrapolating these effects to other retinal degeneration models concurring with neuro-inflammation in the course of disease progression.i.e. retinitis pigmentosa, among others.

Our encouraging results suggest that *in vivo* studies should be conducted with these chemical compounds to evaluate their ability to prevent and/or slow the rate of progression of DR.

## Conclusions

**Figure 7:**
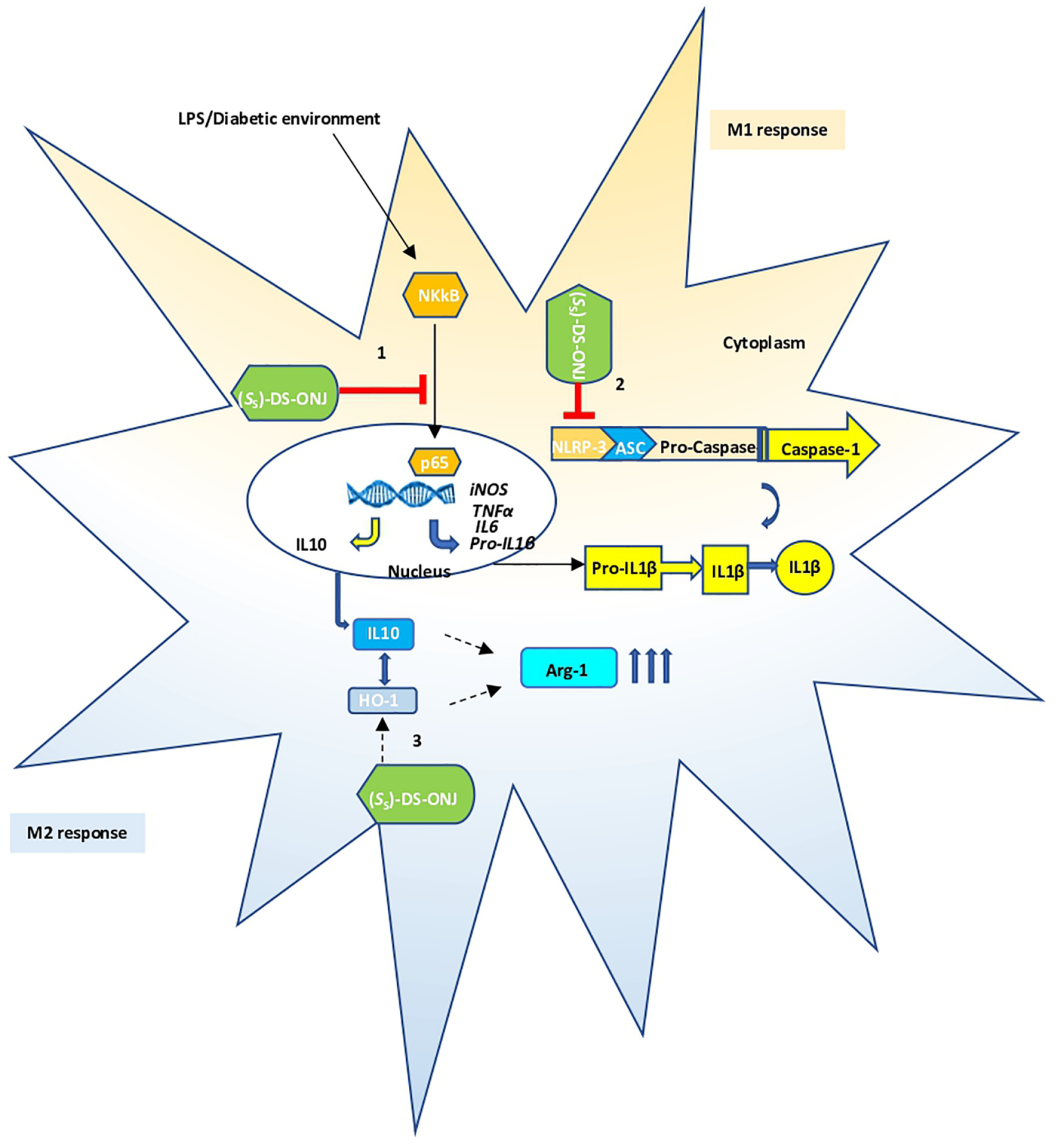
Schematic representation of the anti-neuro-inflammatory mechanisms triggered by (*S*_S_)-DS-ONJ; 1) The pro-inflammatory environment activates NF-κB in microglia cells resulting in the transcription of IL-1β; 2) The diabetic status increases the formation of NLRP3 inflammasome, promotes the cleavage of pro-caspase-1 to its active form and increases IL-1β processing and secretion. The (*S*_S_)-DS-ONJ compound could inhibit NF-κB as well as NLRP3; 3) (S_S_)-DS-ONJ induces the expression of IL-10, HO-1 and arginase-1 driving a switch of microglia polarization towards the M2 stage to counteract inflammation in microglia cells.

In summary, the results obtained in this study demonstrate the anti-inflammatory effect of the (*S*_S_)-DS-ONJ compound in microglial cells and in retinal explants from a classical animal model of T1DM with DR. As such, the field of sp^2^-IGL as DR treatment candidates becomes considerably expanded (**Figure 7**). (*S*_S_)-DS-ONJ treatment upregulates HO-1, IL10 and Arginase-1 expression in microglial cells, and increases their anti-inflammatory M2 response. Our study further highlights the critical involvement of a pro-inflammatory status in DR progression, and strongly suggests that remodeling M1 towards an M2 microglia polarization response represents a hopeful therapeutic option to delay and/or prevent the loss of visual function associated with DR.

## Data Availability Statement

The original contributions presented in the study are included in the article/**Supplementary Material**. Further inquiries can be directed to the corresponding authors.

## Ethics Statement

The animal study was reviewed and approved by Comité de Ética para la Investigación de Cádiz.

## Author Contributions

FC-C generated the experimental data. EA-E generated the experimental data. LG-J drafted and reviewed the manuscript. MI drafted and reviewed the manuscript. EMSF generated the experimental data and drafted and reviewed the manuscript. COM generated funding and drafted and reviewed the manuscript. JMGF generated funding and experimental data. AIA generated funding, drafted and reviewed the manuscript. AMV generated funding and drafted and reviewed the manuscript. AC-C drafted and reviewed the manuscript. CL-T drafted and reviewed the manuscript. MA-D generated funding, drafted and reviewed the manuscript. EMSF and AIA take responsibility for the overall integrity of the study. All authors contributed to the article and approved the submitted version.

## Funding

AIA was supported by grants from Instituto de Salud Carlos III (PI18/01287), Consejería de Salud de la Junta de Andalucía (PI-0123-2018) and Convocatoria de Subvenciones para la Financiación de la Investigación y la Innovación Biomédica y en Ciencias de la Salud en el Marco de la Iniciativa Territorial Integrada 2014-2020 para la Provincia de Cádiz, Fondos ITI-FEDER (PI-0012-2019). MA-D was supported by grants from Convocatoria de Subvenciones para la Financiación de la Investigación y la Innovación Biomédica y en Ciencias de la Salud en el Marco de la Iniciativa Territorial Integrada 2014-2020 para la Provincia de Cádiz, Fondos ITI-FEDER (PI-0029- 2017) and from Instituto de Salud Carlos III (PI18/01287). COM acknowledges funding grant PID2019-105858RB-I00 (MICINN-AEI-FEDER, UE). JMGF acknowledges funding grant RTI2018-097609-B-C21 (MICIU-AEI-FEDER, UE). AMV was funded by grants RTI2018-094052-B-100 (MCIU/AEI/FEDER, UE), S2017/BMD-3684 (Comunidad de Madrid, Spain) and CIBERDEM (ISCIII, Spain).

## Conflict of Interest

The authors declare that the research was conducted in the absence of any commercial or financial relationships that could be construed as a potential conflict of interest.
