# Supplementary material for: Anti-Inflammatory (M2) Response Is Induced by a sp2-Iminosugar Glycolipid Sulfoxide in Diabetic Retinopathy
Source: Front Immunol. 2021 Mar 18;12:632132. doi: 10.3389/fimmu.2021.632132 (PMC8013727; doi:10.3389/fimmu.2021.632132)
Supplement: Supplementary file 1 [file Image_1.pdf]

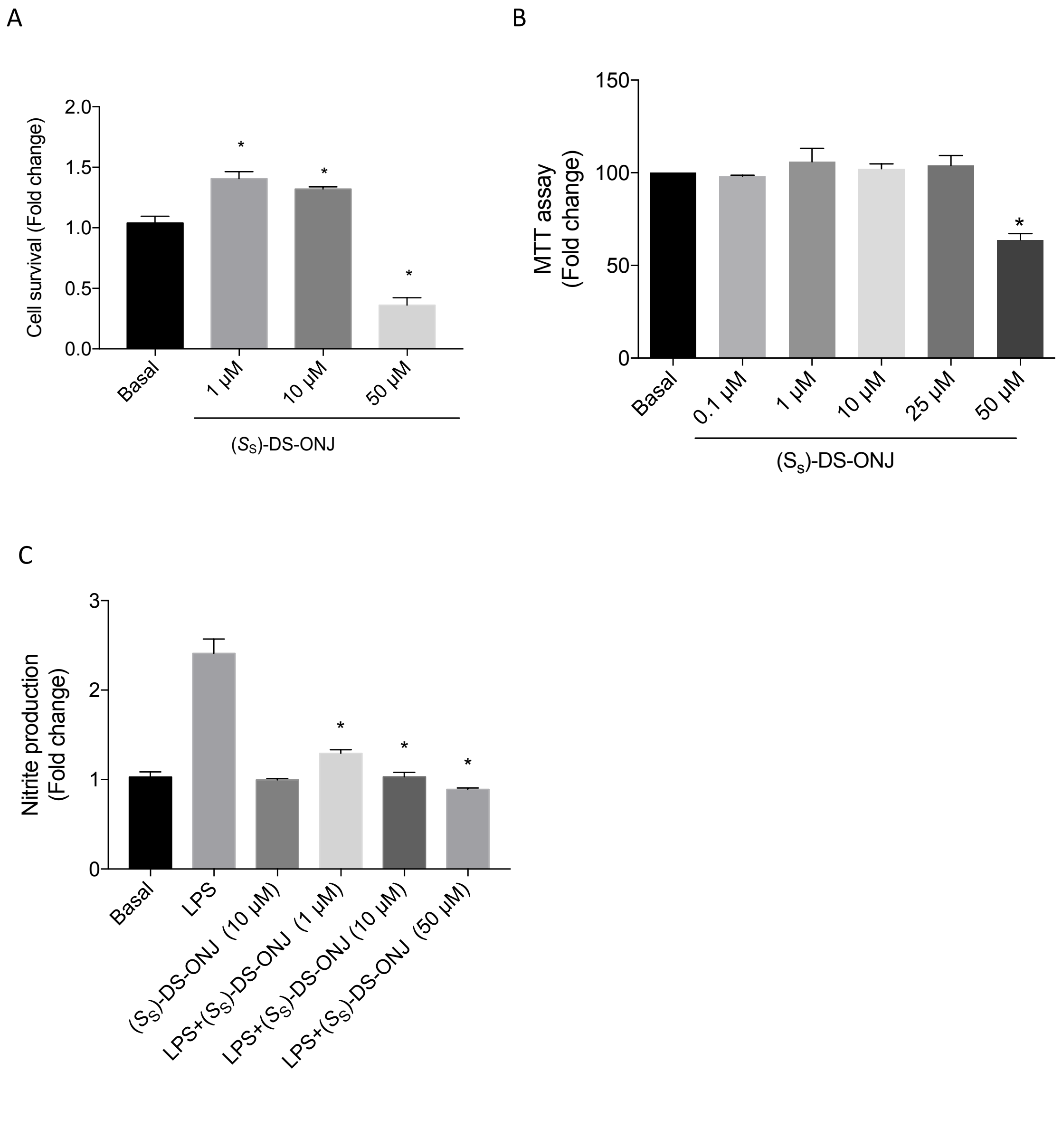

Supplementary Figure 1: Effect of (S_S)-DS-ONJ dose-response in the cellular viability and nitrites production in Bv.2 microglial cells. Bv.2 microglial cells were treated for 24 h with (S_S)-DS-ONJ (1–50 µM) viability was determined by A) crystal violet staining and B) MTT assay. B) Bv.2 microglial cells were treated for 24 h with LPS (200 ng/mL) or LPS plus (S_S)-DS-ONJ (1–50 µM). The results are presented as mean ± SEM (n = 5 independent experiments). The fold changes are presented as relative to the basal value. *$p \leq 0.05$ *vs* LPS treatment (two-way ANOVA followed by Bonferroni t-test).
