# Supplementary material for: Anti-Inflammatory (M2) Response Is Induced by a sp2-Iminosugar Glycolipid Sulfoxide in Diabetic Retinopathy
Source: Front Immunol. 2021 Mar 18;12:632132. doi: 10.3389/fimmu.2021.632132 (PMC8013727; doi:10.3389/fimmu.2021.632132)
Supplement: Supplementary file 2 [file Image_2.pdf]

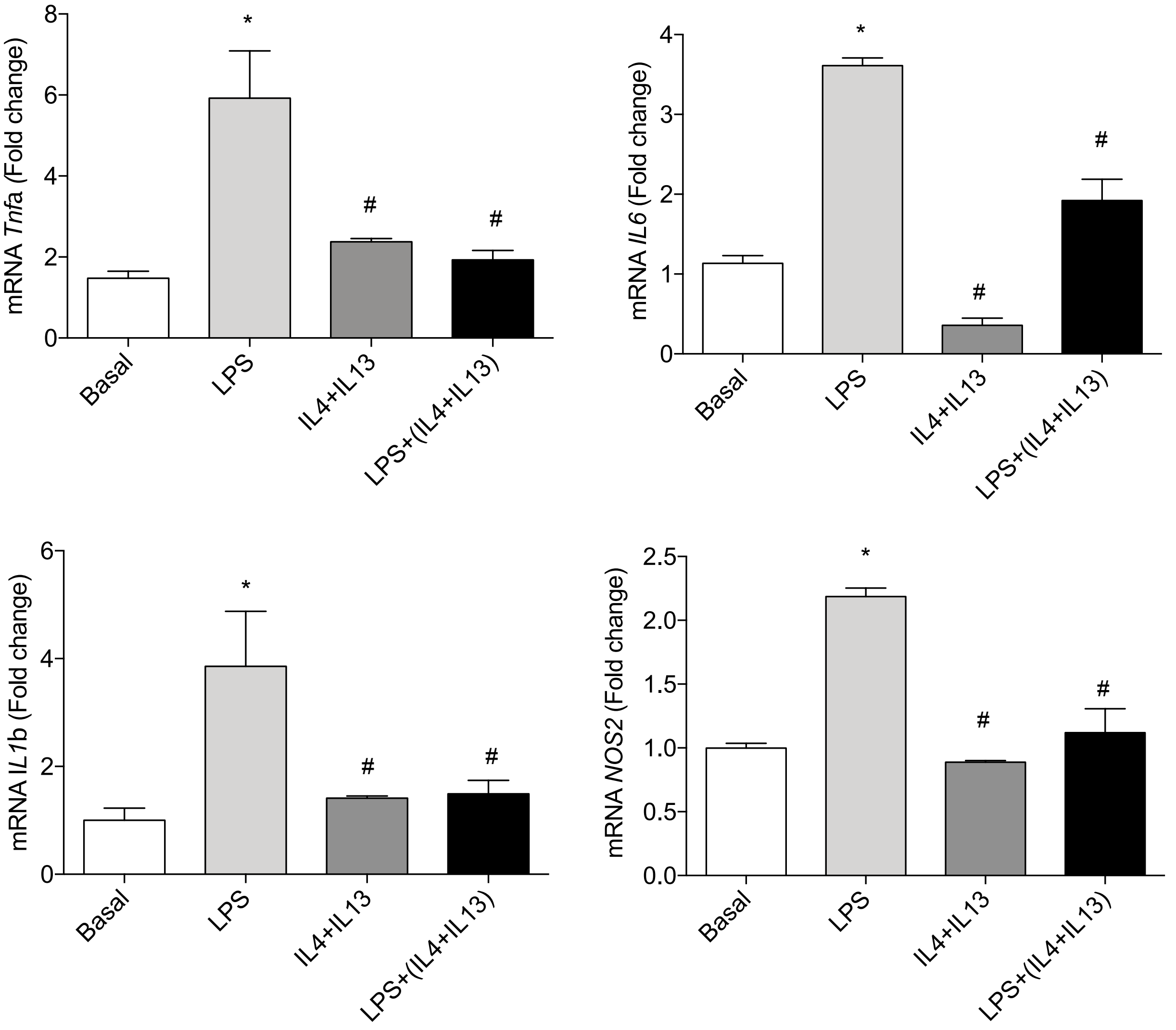

Supplementary Figure 2: Bv.2 microglial cells were treated for 24 h with LPS (200 ng/mL) or LPS plus IL4+IL13 mRNA of *Nos2, Tnfa, Il1b,* and *Il6* mRNA was determined by qRT-PCR. The results are presented as means ± SEM (n = 6 independent experiments). Fold changes are calculated relative to the basal value. *p ≤ 0.05 *vs* Basal treatment, $^{\#}$p ≤ 0.05 *vs* LPS value (two-way ANOVA followed by Bonferroni t-test.)
